# Efflux *MexAB*-Mediated Resistance in *P. aeruginosa* Isolated from Patients with Healthcare Associated Infections

**DOI:** 10.3390/pathogens9060471

**Published:** 2020-06-15

**Authors:** Rania M. Kishk, Mohamed O. Abdalla, Abdullah A. Hashish, Nader A. Nemr, Nihal El Nahhas, Saad Alkahtani, Mohamed M. Abdel-Daim, Safaa M. Kishk

**Affiliations:** 1 Department of Microbiology and Immunology, Faculty of Medicine, Suez Canal University, Ismailia 41522, Egypt; 2 Department of Clinical Pathology, Faculty of Medicine, Suez Canal University, Ismailia 41522, Egypt; mosamaali@hotmail.com (M.O.A.); aahashish80@gmail.com (A.A.H.); 3 Endemic and Infectious Diseases, Faculty of Medicine, Suez Canal University, Ismailia 41522, Egypt; nadernemr@med.suez.edu.eg; 4 Department of Botany, Faculty of Science, Alexandria University, Moharram baik, Alexandria 21515, Egypt; nihal.elnahhas@alexu.edu.eg; 5 Department of Zoology, College of Science, King Saud University, P.O. Box 2455, Riyadh 11451, Saudi Arabia; salkahtani@ksu.edu.sa (S.A.); abdeldaim.m@vet.suez.edu.eg (M.M.A.-D.); 6 Department of Pharmacology, Faculty of Veterinary Medicine, Suez Canal University, Ismailia 41522, Egypt; 7 Department of Medicinal Chemistry, Faculty of Pharmacy, Suez Canal University, Ismailia 41522, Egypt; safaa_keshk@pharm.suez.edu.eg

**Keywords:** efflux pump, MexAB, *Pseudomonas aeruginosa*, HAIs, Egypt, molecular modeling

## Abstract

Today, one of the most important challenges for physicians is the adequate treatment of infections due to multidrug resistant organism (MDR). *Pseudomonas aeruginosa* is considered an opportunistic organism causing different types of healthcare associated infections (HAIs). We aimed to investigate the MDR and pandrug resistance (PDR) rate in *P. aeruginosa* in our region and detect efflux-pump *mexAB* genes and the proposed binding interactions of five different categories of antimicrobial agents with the *mexB* pump. A total of 180 non-duplicated *P. aeruginosa strains* were isolated from patients with HAIs in the Suez Canal University Hospital. Phenotypically, minimum inhibitory concentration (MIC) was done for all MDR and PDR strains before and after addition of efflux pump inhibitor carbonyl cyanide m-chlorophenyl hydrazone (CCCP). Molecular detection of *mexA* and *mexB* genes was done by using polymerase chain reaction (PCR). Most of the isolated strains (126 strains) were MDR (70%); only 10 samples (5.5%) were PDR. *MexA* and *mexB* genes were detected in 88.2% (120 strains) and 70.5% (96 strains) of stains, respectively. All PDR strains (10 stains) carried both *mexA* and *mexB* genes. Efflux *mexAB* genes were detected in all MDR and PDR strains (136 strains). Molecular modeling studies were performed to investigate the modes of intermolecular binding interactions between the antimicrobial agents and *mexB* key amino acids that resulted in MDR and PDR. The current study reported high prevalence of MDR and PDR *P. aeruginosa* in patients with HAIs in the Suez Canal University Hospitals.

## 1. Introduction

One of the important pathogens reported in community and healthcare-associated infections (HAIs) is *P. aeruginosa* [1,2]. The emergence of pandrug-resistant (PDR) and multidrug-resistant strains (MDR) in this organism is of considerable concern, as limited antimicrobial drugs are effective against these resistant strains [1,3]. This phenomenon can be explained by the intrinsic resistance to many antimicrobials due to the presence of efflux transporters with low outer membrane permeability [4,5]. The extrusion of toxic compounds from the cell are promoted through these membrane-associated active transporters. These extruded compounds include antibiotics [6,7,8]. MDR isolates display resistance to three categories of drugs used as anti-*Pseudomonas*, while all types of antibiotics show no effect in the treatment of PDR isolates [9].

Gram-negative bacteria have multidrug efflux systems named resistance-nodulation–cell division (RND) that are clinically significant. The RND-type multidrug efflux systems are not equally expressed in all Gram-negative bacteria. For example; *mexAB*-*oprM*, *mexCD*-*oprJ*, *mexEF oprN* and *mexXY* are more expressed in *P. aeruginosa* [10,11,12]. The RND-type efflux pump system composed of three-component systems. These three components systems are known as proton motive force (*mexD*, *mexB*, *mexY* and *mexF*), outer membrane factors (*oprM*, *oprJ*, *oprN* and OMF) and periplasmic membrane fusion proteins (*mexA*, MFP, *mexX*, *mexC* and *mexF*) [11,12,13,14]. Overproduction of *mexAB*-*oprM* in *P. aeruginosa* plays a significant role in development of MDR strains [15].

Recent studies reported a relationship between *mexXY* system and aminoglycoside resistance in *P. aeruginosa* clinical isolates [16,17]. The most considerable mechanism of resistance is the upregulation of *mexXY* pump [18]. In cystic fibrosis, upregulation of *mexXY* pump seems to be the major contributing factor of *P. aeruginosa* isolates’ resistance to aminoglycosides [1]; (P) *aeruginosa* has been recognized as the foremost pathogen causing HAIs especially at surgical sites and burn wounds as it can colonize inside the injured tissues and flourishes in moist burn wound surfaces [19].

Efflux pump inhibition can be attained by interference with the regulatory mechanisms for the efflux pump expression, blocking of outer pores causing antibiotics efflux or changing the antibiotics structure chemically. Other mechanisms including disturbance of the efflux pump-components assembly or interference with the energy required for the pump activity [20,21]. Efflux pump inhibitors (EPIs) are important for a successful therapy and are used to decrease the level of resistance and increase the intracellular concentration of the therapeutic drugs. The main issue challenged in the production of the EPIs is their toxicity [22]. Carbonyl cyanide m-chlorophenylhydrazone (CCCP) was used as one of the EPIs for *P. aeruginosa* infections by its oxidative phosphorylation action which reduces the ATP production and increases the bacterial membrane permeability by interfering with proton motive force [23].

The X-ray crystallography of tripartite structures has not succeeded although the crystal structures of each components were identified [24]. Difficulty in drugs identification co-crystallized structures in MDR pumps may be due to multisite drug binding and drug oscillation between binding sites during transport [25].

There are two distinct pockets in MDR pumps for the multisite drug-binding: the proximal binding pocket (PBP) and distal binding pocket (DBP) [26]. In the DBP, there is a specific inhibitor-binding hydrophobic pit and it was reported the low-molecular-mass drugs (LMMDs) prefer binding to DBP [26]. *MexAB* has a broad substrate specificity and the binding mode of the studied antibiotics is still unclear, and none of the antibiotics under investigation have been tested by molecular docking. The present study aimed to investigate *P. aeruginosa* MDR and PDR rate and to detect the efflux pump *mexAB* genes as a possible mechanism involved in resistance in our region. Exploring the drug-bound structures of *mexB* from *P. aeruginosa* with five different categories of antimicrobial agents; cephalosporins (ceftazidime), aminoglycosides (gentamicin), monobactam (aztreonam), quinolone (ciprofloxacin) and β-lactam antibiotic (imipenem) and comparing the binding modes with a high-molecular mass compound Lauryl Maltose Neopentyl Glycol (LMNG); which has a competitive inhibitory activity to *mexB* [27], helps in determining the key amino acids that are responsible for the resistant activity.

## 2. Results

**Figure 1:**
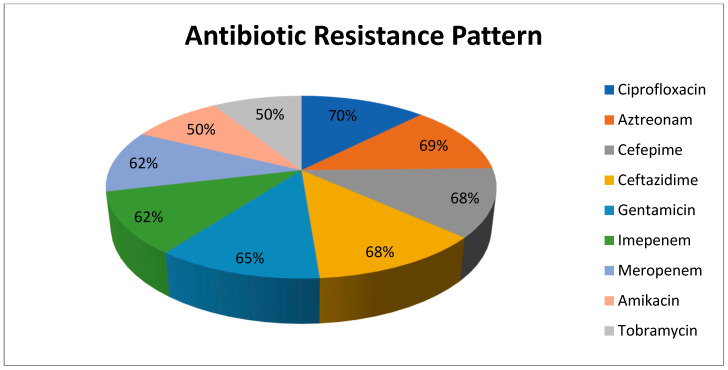
Antibiotic resistance pattern of *P. aeruginosa* isolates (No. 180 strains).

A total of 180 non-duplicated *P. aeruginosa* were isolated from patients with HAIs in the Suez Canal University Hospital. Most of isolates were isolated from burn unit (30%), surgical wards (25%), intensive care units (ICU) (20%), neonatal intensive care unit (NICU) (12%), urology department (8%) and medicine departments (5%). The highest resistance was noticed against ciprofloxacin (70%), aztreonam (69%), cefepime (68%), ceftazidime (68%), gentamicin (65%), imipenem (62%) and meropenem (62%). Half strains were resistant to amikacin (50%) and tobramycin (50%). The highest resistance among MDR strains was to aztreonam (93%) and cefepime (91%) (Figure 1). Out of 180 *P. aeruginosa* isolates, 126 isolates were MDR (70%) and only 10 (5.5%) were PDR.

We noticed that MDR strains (No.126) were isolated from the burn units, surgical wards, ICU and NICU (54.8%, 31.7%, 10.3%, 3.2%, respectively). Half of the PDR strains were isolated from the burn unit (50%, 5 strains) and surgical wards (30%, 3 strains), the remaining 2 strains were isolated from ICU.

The preliminary results of ciprofloxacin susceptibility test, in MDR and PDR strains, using the disk agar diffusion method, showed that 120 isolates (88.2%) were resistant. MIC for ciprofloxacin ranged from 0.25 to 256 mg/L. According to the established breakpoint values recommended by CLSI, the *P. aeruginosa* isolates with MIC ≥ 4 mg/L are considered as ciprofloxacin resistant. Nearly, all tested isolates were resistant to ciprofloxacin by MIC test (MIC ≥ 4 mg/L). By using CCCP as EPI, the MICs of ciprofloxacin for 100 isolates (83.3%) of the ciprofloxacin resistant isolate decreased (more than 4-fold) on the CCCP-supplemented plate. In addition, in other synergy tests by using CCCP and other antibiotics as imipenem, we observed a reduction in MIC on addition CCCP which proved the role of efflux pumps in the resistance to these two drugs (*p* = 0.001). On the other hand, there was no significant change in the MIC of cefotaxime and gentamicin for all the isolates after CCCP addition (*p* > 0.005).

**Figure 2:**
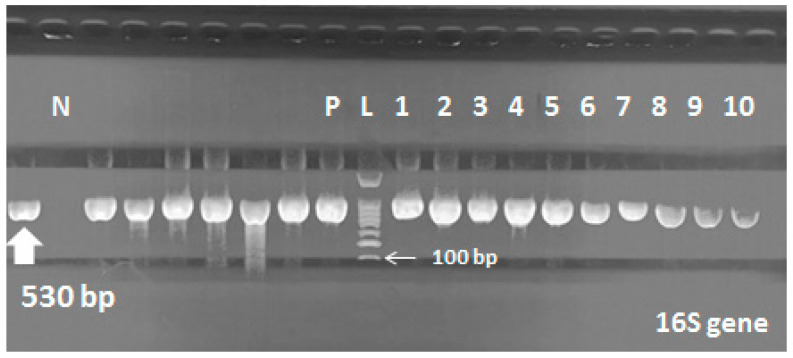
16S gene in *P. aeruginosa*; Lane (L) shows 100-bp molecular size ladder, lane (P) is the positive control, lane (N) is the negative control (*E. coli*), lanes 1 to 10 are the positive samples carrying 16S gene (530 bp).

**Figure 3:**
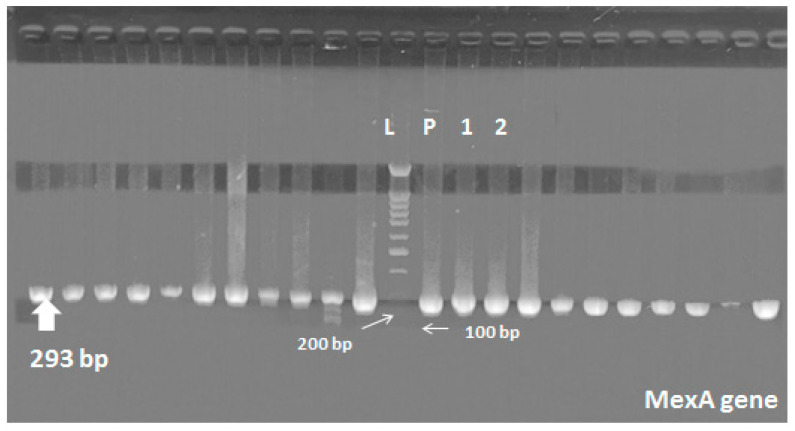
*MexA* gene in *P. aeruginosa*; Lane (L) shows 100-bp molecular size ladder, lane (P) is the positive control, lanes 1 and 2 are the positive samples carrying *mexA* gene (293 bp).

Molecular detection of the 16s gene, *mexA* and *mexB* genes was done by PCR to all MDR and PDR isolates (136 stains). All strains were positive for *Pseudomonas* 16S gene (Figure 2 and Figure 3). *MexA* and *mexB* genes were detected in 88.2% (120 strains) and 70.5% (96 strains) of stains, respectively. Nearly, 80 strains (58.8%) were carrying both *mexA* and *mexB* genes including all PDR strains (10 stains), which were mostly isolated from burn and surgical departments (48.7% and 37.5%, respectively). On the other hand, 56 strains (41.2%) were carrying either *mexA* or *mexB* genes. Most of them were isolated from ICU, NICU and burn units.

Regarding the antibiotic resistance, we found that the strains carrying both of *mexA* and *mexB* genes were more resistant than strains carrying only one gene (the results were statistically significant, *p* < 0.001). All ciprofloxacin and imipenem resistant strains carried at least one resistant gene.

**Figure 4:**
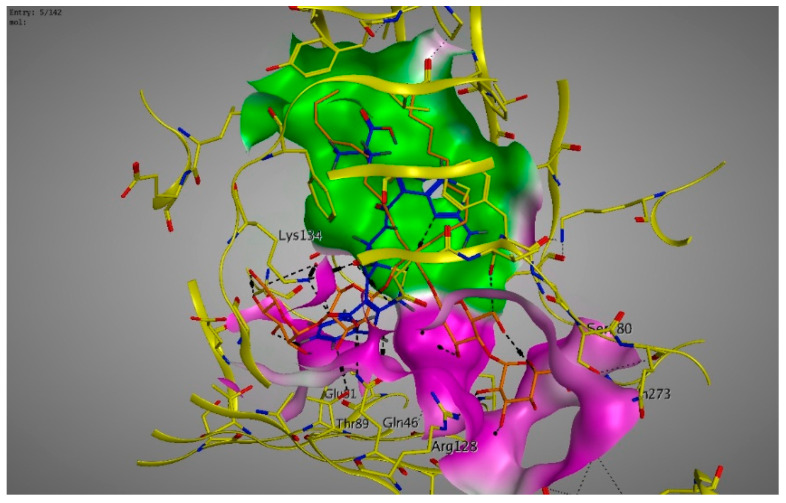
Three-dimensional image of the alignment of the crystal structures of *mexB* (yellow) co-crystallized with LMNG (orange) (PDB 6IIA) and ceftazidime (blue) with comparable positioning observed in the inhibitor-binding hydrophobic pit (green). The carboxylic group was directed to the less hydrophobic part of the active site (magenta).

**Figure 5:**
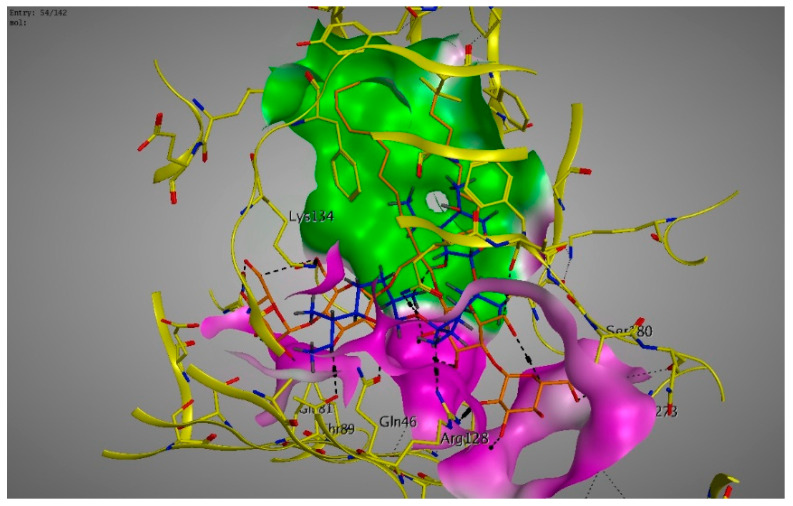
Three-dimensional image of the alignment of the crystal structures of *mexB* (yellow) co-crystallized with LMNG (orange) (PDB 6IIA) and gentamicin (blue) with comparable positioning observed in the inhibitor-binding hydrophobic pit (green). The hydroxy and the amino groups were directed to the less hydrophobic part of the active site (magenta).

**Figure 6:**
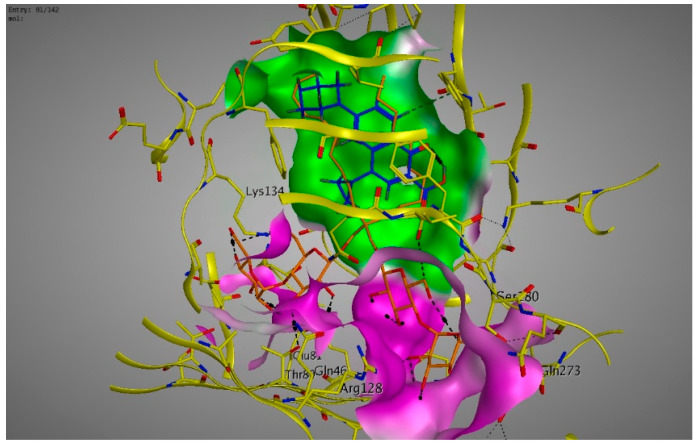
Three-dimensional image of the alignment of the crystal structures of *mexB* (yellow) co-crystallized with LMNG (orange) (PDB 6IIA) and ciprofloxacin (blue) with comparable positioning observed in the inhibitor-binding hydrophobic pit (green).

**Figure 7:**
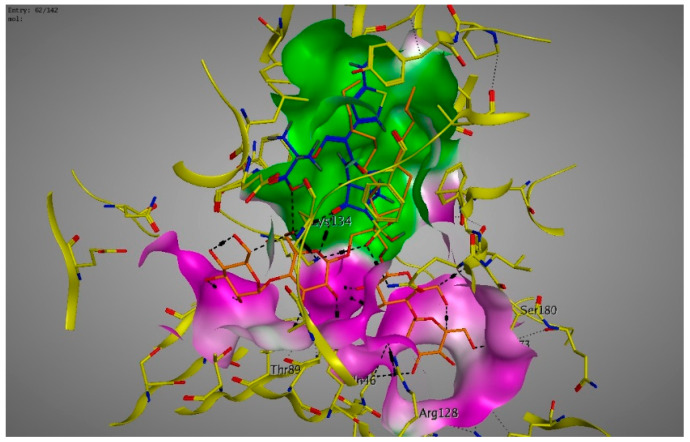
Three-dimensional image of the alignment of the crystal structures of *mexB* (yellow) co-crystallized with LMNG (orange) (PDB 6IIA) and aztreonam (blue) with comparable positioning observed in the inhibitor-binding hydrophobic pit (green).

**Figure 8:**
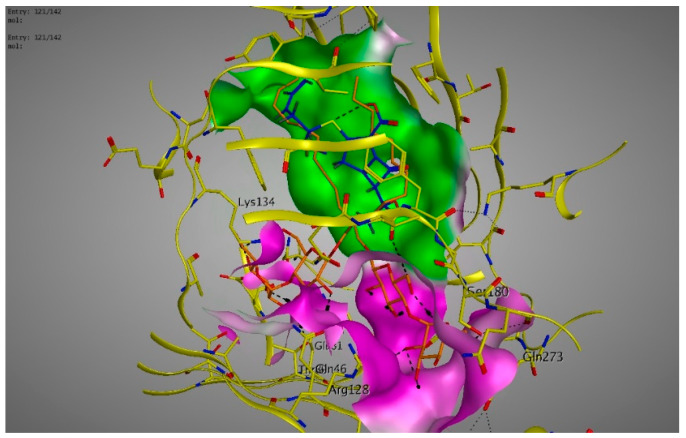
Three-dimensional image of the alignment of the crystal structures of *mexB* (yellow) co-crystallized with LMNG (orange) (PDB 6IIA) and imipenem (blue) with comparable positioning observed in the inhibitor-binding hydrophobic pit (green).

Molecular modeling results showed that the position and binding interactions of ceftazidime, gentamicin, ciprofloxacin, aztreonam and imipenem could be closely replicated as observed with LMNG and *mexB* in the crystal structure PDB 6IIA. The five different categories of antimicrobial agents were bound to the distal binding pocket, where they were inserted into the inhibitor-binding hydrophobic pit showing that both the antimicrobial drugs and the competitive inhibitory LMNG had almost the same binding interactions in the DBP (Figure 4, Figure 5, Figure 6, Figure 7 and Figure 8). The molecular binding modes can illustrate the role of *mexB* in *P. aeriogenosa* resistance to the studied antibiotics.

## 3. Discussions

The emergence of Gram-negative MDR is considered a significant public health issue [28]. The rapid rise of antibacterial resistance is still a major global health problem that can impair the antibacterial agents’ effectiveness and wastes the efforts for developing new drugs. Efflux pumps have a great concern in emergence of *P. aeruginosa* antibacterial resistance [6,7,29].

The present study aimed to study the prevalence of MDR and PDR *P. aeruginosa* and its relationship with the efflux pump *mexAB* genes as a possible factor involved in resistance in the Suez Canal University Hospital, Ismailia, Egypt. Our data showed high prevalence of MDR among the isolated strains (70%), and only (5.5%) of the isolates were PDR. In concordance with our results, a study from Iran published in 2016, reported high MDR rate in *P. aeruginosa* isolates (66%) from burns unit and only one strain was PDR (0.66%) [30].

We noticed increased rates of MDR and PDR in the burns unit (54.8% and 50%, respectively) followed by SSIs in surgical wards (31.7% and 30%, respectively). As a matter of fact, *P. aeruginosa* is the main pathogen causing burn infections and is considered as a major colonizer of burn wounds as the moist surfaces of burn wounds represents a favorable medium for its growth, and because of its ability to persist well in hospital environments [19].

The highest resistance was noticed against ciprofloxacin (70%), aztreonam (69%), cefepime (68%), ceftazidime (68%), gentamicin (65%), imipenem (62%) and meropenem (62%). A recent study in burn centers of Tehran, Iran, reported high rate of resistance to ciprofloxacin, amikacin and gentamicin (over 85%) [31] which was higher than our findings. This difference may be due to different samples sources, as in our study, we collected specimens from all cases with HAIs not from burns unit only. Burns unit, as a critical care unit, is rich in bacteria with severe antimicrobial resistance. This explains the high rates of MDR and PDR in burns unit in our study.

We noticed that more than 80% of the MDR isolates were resistant to more than seven antibiotics and more than 60% of the isolates were resistant to more than ten antibiotics. In a similar study, Pellegrino et al. reported lower percentage to co-resistance to seven or ten antibiotics, at two different centers (nearly 32% and 40%, respectively) [32]. The lower resistance rate of that study compared to ours is expected as the antibiotic resistance rates has increased radically all over the world in the past few years. On the other hand, Delpano et al. reported higher antibiotic resistance compared to our study that 100% of the isolates were resistant to ciprofloxacin, tobramycin, gentamicin, cefepime, imipenem and meropenem [33].

Overexpression of efflux pumps could be the leading cause of MDR in bacteria as it leads to a decreased intracellular concentration of antibiotics and reduced susceptibility to antimicrobial agents due to continuous expelling of structurally unrelated drugs [34]. This explains the over representation of *mexA* and *mexB* genes (70.5% and 88.2%, respectively) in our study. We noticed that all PDR isolates were carrying both *mexAB* genes. This over-representation of *mexAB* genes is matched with the fact that the antibiotics that are substrates of the corresponding transporters are used usually in patient treatment plans.

Molecular docking was done using the molecular operating environment (MOE) program, to investigate the intermolecular binding affinity between the studied antimicrobial agents and *mexB* which was one of the overexpressed efflux pumps in our study. This binding affinity is directly correlated to the antimicrobial resistance due to the efflux mechanisms which result in a decreased concentration of the antimicrobial agents intracellularly. Some of the key amino acid residues that had intermolecular bindings with all of the antimicrobial agents were Lys134, Gln46 and Gln176.

**Figure 9:**
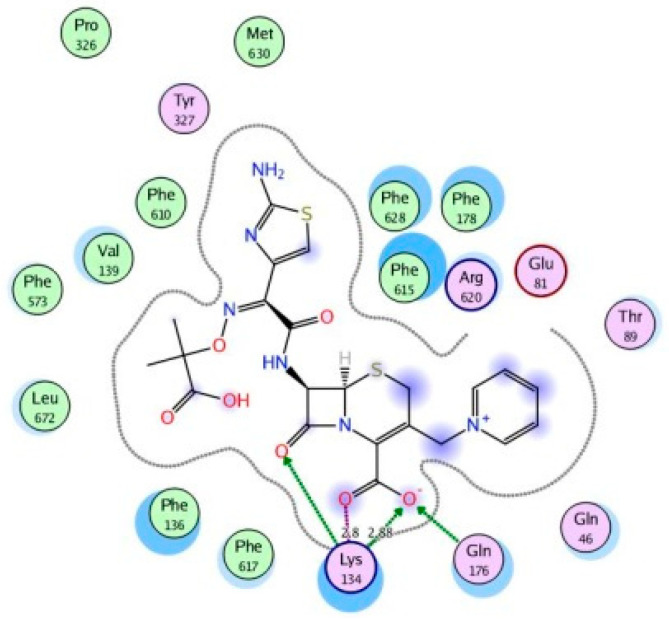
Two-dimensional image of the binding interactions of ceftazidime in the *mexB* active site with Lys134, Gln46, Gln176, Phe178 as key binding amino acids.

For the cephalosporin ceftazidime, the 2-aminothiazol-4-yl group elongated parallel in the space above the inhibitor-binding pit, mimicking the LMNG positioning. The ceftazidime carboxylate formed strong ionic bonding with the basic amino acid Lys134. H-bonds were formed with Lys134, Gln46 and Gln176 in addition to arene-arene bindings between thiazole ring and Phe178 in the hydrophobic pit (Figure 9).

**Figure 10:**
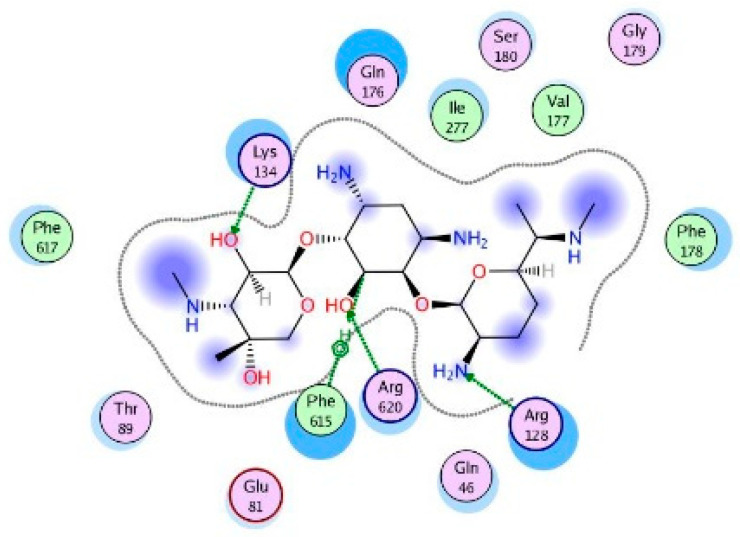
Two-dimensional image of the binding interactions of gentamicin in the *mexB* active site with Lys134, Gln46, Gln176, Arg128, Arg620 and Phe615 as key binding amino acids.

The aminoglycoside gentamicin was flexibly bound towards the exit and the entrance of DBP, forming H-bonds with Arg128, Lys134, gln176 and Arg620. Arene–H binding was also formed with Phe615 (Figure 10).

**Figure 11:**
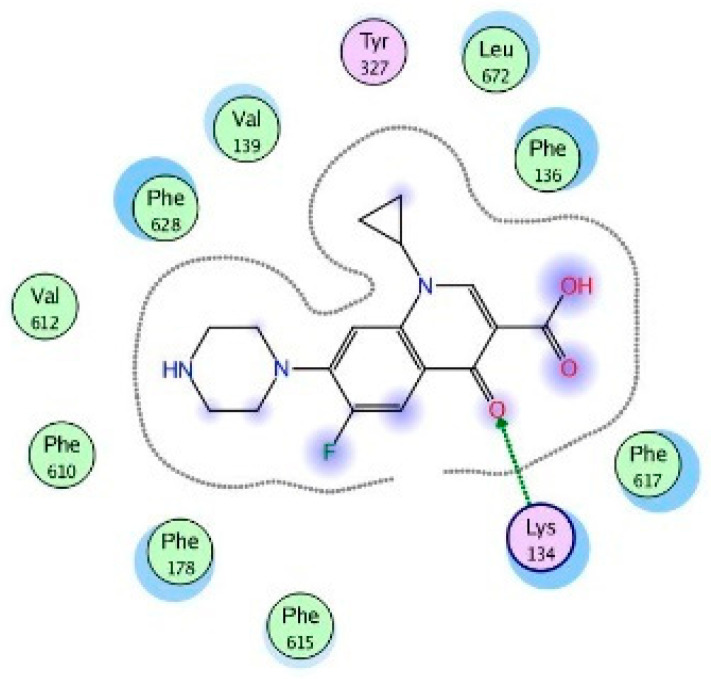
Two-dimensional image of the binding interactions of ciprofloxacin in the *mexB* active site with Phe628, Phe178, Phe610, Arg620 and Arg128 as key binding amino acids.

The quinolone ciprofloxacin was mainly inserted in the inhibitor-binding pit and formed arene-arene bindings with Phe628 and Phe178, arene–H bindings with Phe610 and Phe178. H-bonds were also observed with Arg620 and Arg128 (Figure 11).

**Figure 12:**
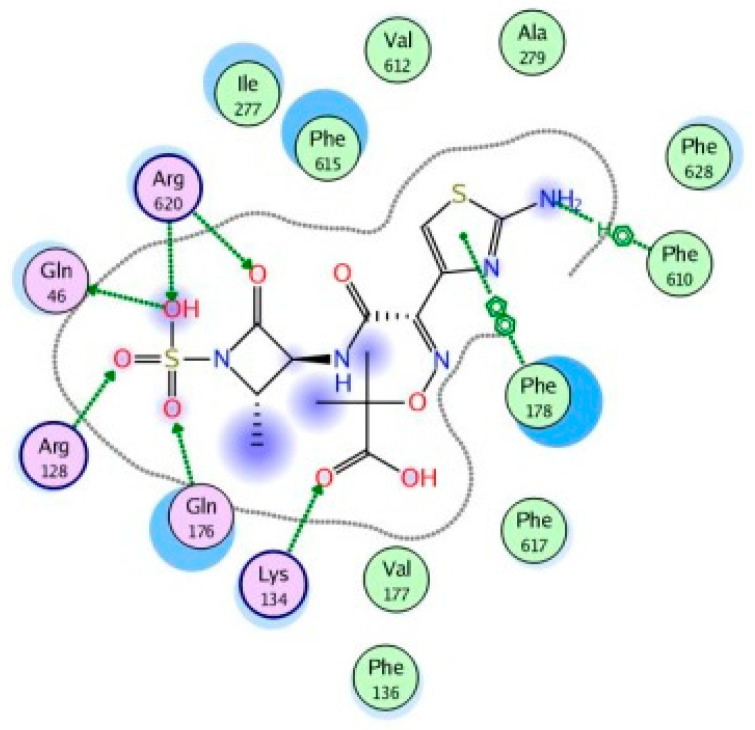
Two-dimensional image of the binding interactions of aztreonam in the *mexB* active site with Arg128, Lys134, gln176, Gln46, Lys151, Ser87, Arg620, Phe610 and Phe178 as key binding amino acids.

The monobactam aztreonam was flexibly bound towards the exit and the entrance of DBP, forming H-bonds with Arg128, Lys134, gln176, Gln46, Lys151, Ser87 and Arg620. Arene–H binding was also formed with Phe610 and arene-arene binding with Phe178 (Figure 12).

**Figure 13:**
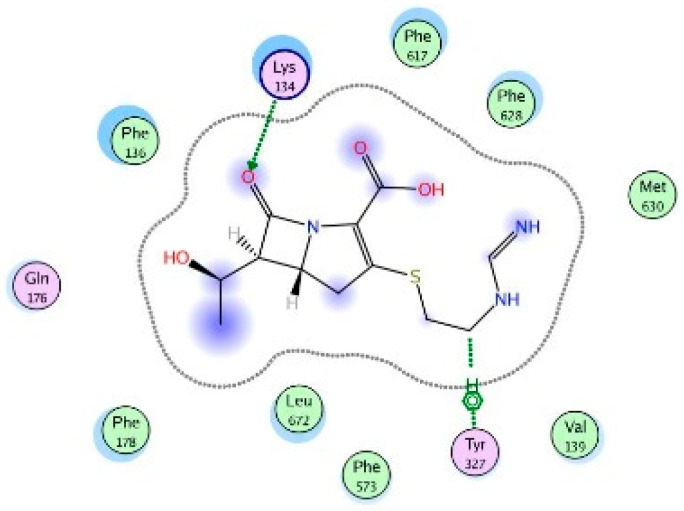
Two-dimensional image of the binding interactions of imipenem in the *mexB* active site with Arg128, Lys134, Phe610 and Phe628 as key binding amino acids.

Finally, the β-lactam imipenem formed H-bonds with Lys134 and Arg128 in addition to arene–H bindings with Phe610 and Phe628 in the hydrophobic pit (Figure 13).

The limitation of this study:

The presented data showed the presence of the genes in the genomes and provide no insight into the level of their expression. Further studies are needed to analyze conservation of the genes sequence in the genomes (their presence) of diverse clinical isolates and correlate it (their presence) with antibiotic resistance. Our study also did not discuss the correlation between the presence of single or both *mexAB* and MDR phenotype as we did not detect *mexAB* in non MDR isolates by PCR.

## 4. Materials and Methods

This cross-sectional descriptive study was carried out during the period from January 2019 to December 2019. A total of 180 strains of *P. aeruginosa* were isolated from patients with Healthcare Associated Infections in the Suez Canal University Hospital. Samples were processed in microbiology laboratory, Faculty of Medicine, Suez Canal University.

### 4.1. Bacterial Isolation and Identification

The collected samples were cultured on blood, MacConkey agar, cetrimide agar (Oxoid, UK) and *Pseudomonas* P agar media. Colonies were identified as *P. aeruginosa* by colony morphology, Gram stain and different biochemical reactions [35]; (P) *aeruginosa* ATCC 27853 was used as a reference strain. The isolates were preserved at −80 °C in glycerol 15% in brain heart infusion broth (BHIB, Oxoid, Basingstoke, UK) and then subculturing in BHIB at 37 °C for 24 h.

### 4.2. Antimicrobial Susceptibility Testing

Antibiotic susceptibility testing was performed by Kirby–Bauer disk diffusion method and interpreted according to the Clinical Laboratory Standard Institute (CLSI) guidelines [36]. The antibiotics tested were ciprofloxacin, aztreonam, cefepime, tobramycin, ceftazidime, gentamicin, amikacin, imipenem and meropenem (Oxoid, Basingstoke, UK). Interpretation of susceptibility testing was performed according to CLSI [36].

Multidrug resistant organisms (MDR) were defined as non-susceptibility (i.e., resistant or intermediate) to at least one agent in at least 3 antimicrobial classes of the following 5 classes: cephalosporins (cefepime, ceftazidime), β-lactam/β-lactam β-lactamase inhibitor combination (piperacillin, piperacillin/tazobactam), carbapenems (imipenem, meropenem, doripenem), fluoroquinolones (ciprofloxacin or levofloxacin) or aminoglycosides (gentamicin, tobramycin or amikacin) (CDC, 2020). Otherwise, Pan-drug resistant (PDR) *Pseudomonas* expressed resistance to all antibiotics [9].

### 4.3. Role of Efflux Pump in Antibiotic Resistance

The MICs of ciprofloxacin, imipenem, ceftazidime and gentamicin were evaluated for all MDR *and* PDR *P. aeruginosa* isolates. Then, CCCP was added in a concentration of 10 μM to each Mueller-Hinton agar plates containing 0.5 to 128 µg/mL concentration of each antibiotic used. The CCCP final concentration in the Mueller-Hinton agar was 25 µg/mL. Then, MIC was repeated. A plate with CCCP and no antibiotics was used as control. The reduction in MIC at least 4-fold of any antibiotics with CCCP indicates the presence of efflux pump in isolates [37].

### 4.4. Molecular Detection of mexA and mexB Genes by PCR

DNA extraction was done using Qiagen DNA Mini kit 51,304. We used primers for detection of 16s gene. PCR conditions were adjusted as described by Pirnay and his colleagues [38]. *MexA* and *mexB* genes were detected as described before [39]. The PCR products were analyzed by agarose gel electrophoresis on 1.5% agarose (w/vol.) containing 0.5-mg/mL ethidium bromide (Qiagen, Germany) using a 100-bp DNA ladder was used as the size marker (Roche, Germany). Reaction mixtures without a DNA template served as negative controls. All primers and product lengths are shown in Table 1.

### 4.5. Statistical Analysis

All statistical analyses were performed using the statistical package for social sciences program (SPSS version 22 for windows). Statistical significance was considered at *p*-value ≤ 0.05.

### 4.6. Molecular Modeling and Docking

Docking studies were performed using the molecular operating environment (MOE) software and *mexB* of *P. aeruginosa* co-crystallized with the LMNG (PDB 6IIA) [40,41]. All minimizations were done until an RMSD gradient of 0.01 kcal/mol/A was obtained. The MMFF94 forcefield and partial charges were automatically calculated. The alpha triangle placement was chosen to determine the poses. The free energy of ligand binding from a given pose was estimated by the London ΔG scoring function. Refined results were rescored using the London ΔG scoring function. The output database dock file was created with different poses for each ligand and arranged according to the final score function (S).

## 5. Conclusions

In conclusion, the current study recognized the high MDR and PDR *P. aeruginosa* rates in burn and wound samples obtained from the Suez Canal University Hospital. In all MDR and PDR isolated strains Efflux *mexAB* genes were detected. The studied binding interactions of different drug categories help in the molecular study of the *mexB*. The molecular modeling could show the key amino acids in *mexB* that interact with several antimicrobials. The studied docking can give a promising investigation for a future treatment for the resistant *P. aeruginosa* strains.

## Figures and Tables

**Table 1:** Primers sequences used in this study.

Primer	Sequence	Amplified Product
16s gene		
Forward	5′ATGGAAATGCTGAAATTCGGC 3′	530 bp
Reverse	5′CTTCTTCAGCTCGACGCGACG 3′	
MexA gene		
Forward	5′CGACCAGGCCGTGAGCAAGCAGC3′	293 bp
Reverse	5′GGAGACCTTCGCCGCGTTGTCGC 3′	
MexB gene		
Forward	5′GTGTTCGGCTCGCAGTACTC 3′	244 bp
Reverse	5′AACCGTCGGGATTGACCTTG 3′	
